# Simulation of the Fate and Seasonal Variations of **α**-Hexachlorocyclohexane in Lake Chaohu Using a Dynamic Fugacity Model

**DOI:** 10.1100/2012/691539

**Published:** 2012-12-18

**Authors:** Xiang-zhen Kong, Wei He, Ning Qin, Qi-Shuang He, Bin Yang, Huiling Ouyang, Qingmei Wang, Chen Yang, Yujiao Jiang, Fuliu Xu

**Affiliations:** MOE Laboratory for Earth Surface Processes, College of Urban and Environmental Sciences, Peking University, Beijing 100871, China

## Abstract

Fate and seasonal variations of **α**-hexachlorocyclohexane (**α**-HCH) were simulated using a dynamic fugacity model in Lake Chaohu, China. Sensitivity analyses were performed to identify influential parameters and Monte Carlo simulation was conducted to assess model uncertainty. The calculated and measured values of the model were in good agreement except for suspended solids, which might be due to disregarding the plankton in water. The major source of **α**-HCH was an input from atmospheric advection, while the major environmental outputs were atmospheric advection and sediment degradation. The net annual input and output of **α**-HCH were approximately 0.294 t and 0.412 t, respectively. Sediment was an important sink for **α**-HCH. Seasonal patterns in various media were successfully modeled and factors leading to this seasonality were discussed. Sensitivity analysis found that parameters of source and degradation were more important than the other parameters. The sediment was influenced more by various parameters than air and water were. Temperature variation had a greater impact on the dynamics of the model output than other dynamic parameters. Uncertainty analysis showed that the model uncertainty was relatively low but significantly increased in the second half of the simulation period due to the increase in the gas-water diffusion flux variability.

## 1. Introduction

 Organochlorine pesticides (OCPs) have been under increasing scrutiny due to their refractory qualities and high ecotoxicity. Hexachlorocyclohexanes (HCHs), a type of OCPs, have already been listed by the Stockholm Convention on Persistent Organic Pollutants in the first batch of control compounds [1]. During the 1960s and 1970s, there was a substantial amount of production and usage of HCH pesticides in China, resulting in high level of residues in the soil [2]. Through surface runoff, undercurrent, osmosis, leaching, and other transport mechanisms from the soil into the surface water, the water bodies, such as lakes, have also been severely polluted by HCH pesticides. According to historical data, the quantity of emitted OCPs in the Lake Chaohu water bodies amounted to 1.16 tons in 1984. Among the isomers of HCHs, *α*-HCH can cause human neurological disorders and gastrointestinal discomfort, resulting in liver and kidney damage, human endocrine system disorders and immune system abnormalities [1]. Therefore, an understanding of the distribution and dynamics of *α*-HCH in lake environments is extremely crucial.

The multimedia model is a mathematical model developed in the 1980s based on the concept that the physical and chemical properties of environmental systems and pollutants synergistically determine the concentration, distribution, and migration of contaminants throughout the transformation process between environmental compartments [3]. Mackay [4] and Mackay and Paterson [5, 6] proposed a fugacity model to simplify the structure of the multi-media model and the calculation process. This model has been widely used in describing the environmental behaviors of pollutants in global, regional, and local environments [7–9]. There are four levels in the fugacity model. A level IV fugacity model is appropriate when continuous changes in the concentrations of particular pollutants are studied over a period of time [8, 10]. 

Few studies have been conducted that focus on the seasonal variation in *α*-HCH using the level IV fugacity model. In this study, the fate and seasonal variation of *α*-HCH in the air, water, and sediment of Lake Chaohu were examined. Since the usage of industrial HCHs was banned in 1983 and lindane was applied instead [10], the *α*-HCH emissions can be assumed to be zero. The results of this model can reveal the main source, the migration and transformation processes, and the most influential parameters on the fate and seasonal variations of *α*-HCH in the of Lake Chaohu environment. The uncertainty of the model was also assessed using a Monte Carlo simulation.

## 2. Materials and Methods

### 2.1. Model Development

**Figure 1:**
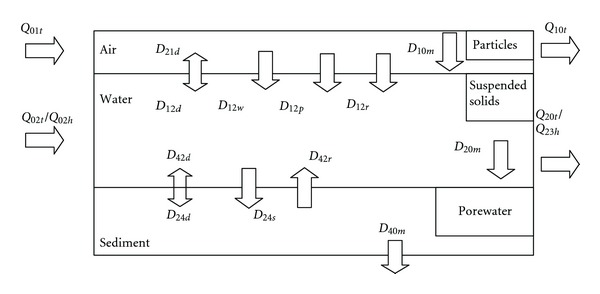
Transport fluxes of *α*-HCH in and out of the Lake Chaohu area and between the adjacent compartments. *D*_12*d*_, *D*_21*d*_, *D*_24*d*_, and *D*_42*d*_ represent the diffusion processes between air/water and water/sediment. *D*_12*p*_ and *D*_12*w*_ represent the dry and wet deposition from air to water, respectively. *D*_12*r*_ represents scavenging by precipitation. *Q*_01*t*_, *Q*_02*t*_, and *Q*_02*h*_ represent the input from air advection, water inflows, and waste water discharge, respectively. *Q*_10*t*_, *Q*_20*t*_, and *Q*_23*h*_ represent the output from air advection, water outflows and water reuse by industry, and agriculture, respectively. *D*_10*m*_,*D*_20*m*_ and *D*_40*m*_ represent the degradation occurring in the air, water, and sediment, respectively.

The framework of the model in this study was based on the quantitative water, air, sediment interaction (QWASI) fugacity model [11], with the major difference being the inclusion of atmospheric advection input and output of the system. This model included three main compartments: atmosphere, water, and sediment, which were represented by the subscripts 1, 2, and 4, respectively. The atmospheric phase was comprised of two subphases: gaseous and particulate matter. The aqueous phase also comprised two sub-phases: water and suspended solids. The sediment phase consisted of porewater and a solid phase. The model framework is shown in Figure 1. The basic characteristics of the model for Lake Chaohu are shown in Table 1.

Model parameter symbols, units, values and data sources are shown in Tables S1 and S2 in supplementary materials available online at doi:10.1100/2012/691539. The model had a total of 46 parameters, including 23 environmental parameters, 12 interface mass transfer parameters, and 11 physicochemical parameters for the pollutant. The environmental parameters included temperature, lake area, height and sub-phase volume fraction determined by the literature or laboratory measurements. The physicochemical parameters, such as the gas constant, Henry's constant, and saturated vapor pressure, were obtained from the literature. The environmental kinetics of the process parameters, including the rate of degradation, the rate of diffusion, migration constant, molecular diffusion path length, atmospheric wet and dry deposition rates, deposition rate, and cleaning coefficients, were obtained from the relevant literature. Fifteen parameters had annually changing values, including the environmental parameters (*h*_2_, *X*_13_, *Q*_01*t*_ *Q*_10*t*_, *Q*_02*t*_, *Q*_20*t*_, *Q*_23*h*_, *Q*_02*h*_, *T*, *C*_1_, and *X*_23_) and the mass transfer parameters (*K*_12_,*K*_21_,*K*_42*r*_, and *K*_*w*_). The parameter *h*_2_ included hourly data; *T*, *K*_12_, *K*_21_, *K*_42*r*_, and *K*_*w*_ had daily data, and *X*_13_, *Q*_01*t*_(*Q*_10*t*_), *Q*_02*t*_, *Q*_20*t*_, *Q*_23*h*_, *Q*_02*h*_, *C*_1_, and *X*_23_ had monthly data. Ot her parameters were used in terms of annual average values, and they remained constant during the simulation. In addition, Henry's constant, saturated vapor pressure, and the fugacity rate of the pollutant were primarily obtained using a temperature of 25°C. The temperature correction required for these parameters and the correction equation are shown in (1) [12]:

(1) log10PT=log10P25+A×(1298−1T+273),

where *P*_*T*_ is the physical and chemical parameter values at *T* (°C) (Henry's constant, the saturation vapor pressure, or fugacity rate); *P*_25_ is the physical and chemical parameters at 25°C; *A* is the temperature correction coefficient (for Henry's constant, saturated vapor pressure, and fugacity rate). To obtain the total river inflows of Lake Chaohu from May 2010 to February 2011, monthly data from May 1987 to April 1988 were collected [13] along with the corresponding daily precipitation data from the China Meteorological Data Sharing Service System [14]. There was a significant linear relationship between the river inflow and the precipitation data. Using this linear relationship and the monthly precipitation data from May 2010 to February 2011 for Lake Chaohu, the river inflow (*Q*_02*t*_) for the simulation period was easily calculated. This calculation was based on the assumption that there were no significant landscape level changes which largely modifies fate of precipitation at catchment area since 1980s. In addition, the values of water inflow were not important to the fate of *α*-HCH in the lake, which will be revealed in the sensitive analysis (Section 3.3). The average monthly river outflow (*Q*_02*t*_) of Lake Chaohu was based on the water balance calculation of inflow and water level in addition to the rates of industrial and agricultural water consumption (*Q*_23*h*_) [13].

Taking into consideration that industrial HCHs were banned in 1983 and lindane (*γ*-HCH) was applied instead [10], emissions of *α*-HCH in the vicinity of Lake Chaohu were assumed to be zero during the simulation. Atmospheric *α*-HCH input originated from atmospheric advection. The *α*-HCH concentration in the advection within the study area (*C*_1_) was determined according to the sampled values on the lake side (four samples in total). The daily average wind speed and direction during the simulation period in Lake Chaohu area were obtained from the China Meteorological Data Sharing Service System [14]. The volumes of atmospheric advections (*Q*_01*t*_, *Q*_10*t*_) were calculated according to the corresponding atmospheric height, the area of Lake Chaohu, and the wind speed. *α*-HCH input originated from water inflows was determined by the summation of the input amount from all the rivers around the lake [15].

The mass transfer coefficients of both sides of the gas-water interface (*K*_12_ and *K*_21_) were calculated according to the method proposed by Southworth [16]. The resuspension coefficient (*K*_42*r*_) was calculated according to the formula from Tu et al. [13]. The specific equations are as follows:

(2) K12=11.375·(WS+RS)·(18MW)0.5,

(3) K21=0.2351·RS0.969·(32MW)0.5ahW0.673(if  WS≤1.9 m/s,a=1;else  a=exp⁡[0.529(WS−1.9)]),

(4) $$\begin{array}{c} K_{42r}=3\times 10^{-8}\cdot \frac{\text{WS}}{h_{W}}, \end{array}$$

where WS is the average wind speed (m/s); RS is the surface flow velocity (m/s); MW is the molecular weight (g/mol); and *h*_*W*_ is the water depth (m).

The transfer and transformation processes defined in the model are shown in Table S3 (Supplementary Material). Details can be found in Mackay and Paterson [17]. The level IV fugacity model can be expressed by (5), where fugacity is symbolized by *f*(Pa). The processes considered in the model included the advection of the air and water phases, diffusion and dry/wet deposition between the air and water, diffusion, sedimentation, and resuspension between the water and sediment, and the degradation process during the main phase. In this study, the fourth-order Runge-Kutta method was applied to solve the differential equations by simulating step of 1-hour. The time period of the dynamic simulation was from May 1, 2010 to February 28, 2011. The seasonal variation of the *α*-HCH concentration for each compartment of the environment was simulated and compared to the measured values. Validation data were obtained from the monthly samples of atmospheric aerosols on an island in the lake, water and suspended solids (four sample sites in total) collected from May 2010 to February 2011 [18, 19] and from the 14 sediment samples collected in August 2008 from Lake Chaohu [20].

(5) V1Z1df1dt =T01t−(D12d+D12p+D12r+D12w+D10m−D10t)f1  +D21df2,V2Z2df2dt =T02t+(D12d+D12p+D12g+D12w)f1  −(D21d+D24d+D24s+D20m+D20t+D23h)f2   +(D42d+D42r)f4,V4Z4df4dt =(D24d+D24s)f2−(D42d+D42r+D40m)f4.

### 2.2. Sensitive Analysis

When a system error of the model cannot be eliminated, the accuracy of the parameters is the most important factor in model accuracy, particularly regarding some sensitive parameters [2]. Therefore, a sensitivity analysis was conducted for all parameters involved in the model (except the gas constant, *R*). For constant parameters, a local sensitivity analysis was applied which implemented a “perturbation” near the best estimate value of a parameter, and the variation of model outputs was studied under the condition that other parameters remained unchanged. The Morris classification screening method, a widely applied local sensitivity analysis method, was used [21]. A variable was selected, and the value changed to the fixed step size, while the other parameters remained the same. The sensitivity index of the parameter was the average of the multiple disturbance calculated Morris coefficient:

(6) $$\begin{array}{c} S=\sum_{i=0}^{n-1}\frac{\left(Y_{i+1}-Y_{i}\right)/Y_{0}}{\left(P_{i+1}-P_{i}\right)/100}/\left(n-1\right), \end{array}$$

where *S* is the Morris coefficient; *Y*_*i*_ is the model output value in the *i*th simulation; *Y*_0_ is the model calculation result when the parameter is set at the initial value; *P*_*i*_ is the percentage change of the parameter value the for *i*th simulation ; and *n* is the number of runs.

 Cao et al. [2] proposed that when the step size is small enough, the nonlinear effects of the parameters of the model output are negligible. In this study, it was assumed that the parameters increased and decreased by 10% on the basis of the original value. *Y*_0.9_, *Y*_0_, and *Y*_1.1_ are the output results when the parameter was multiplied by 0.9, 1 and 1.1, respectively. The sensitivity coefficient (*C*_*s*_) is as follows:

(7) $$\begin{array}{c} C_{s}=\text{Abs}\left(\frac{Y_{1.1}-Y_{0.9}}{0.2\times Y_{0}}\right). \end{array}$$

The effect of the parameters on the model output was not only associated with corresponding *C*_*s*_ values of the parameters but was also related to the fluctuation range of the parameters in the environment [2]. With the same *C*_*s*_ value, those parameters with higher variability have greater impacts on the model than those with lower variability. In this study, the sensitivity coefficient after the correction of the coefficient of variation (*C*_*n*_) for the parameters was also calculated by [2], such that *C*_*n*_ = *C*_*s*_ × CV, where CV is the coefficient of variation of the parameter.

For the dynamic parameters in the model, the dynamic sensitivity coefficient (SCV) is calculated as follows [22]:

(8) $$\begin{array}{c} \text{SC}{\text{V}}_{i}=\frac{\Delta \text{C}{\text{V}}_{i}^{Y}/\text{C}{\text{V}}_{i}^{Y}}{\Delta \text{C}{\text{V}}_{i}^{X}/\text{C}{\text{V}}_{i}^{X}}, \end{array}$$

where CV_*i*_^*X*^ and CV_*i*_^*Y*^ indicate the corresponding coefficients of variation of the *i*th input parameter and the output parameter, respectively, and Δ*CV*_*i*_^*X*^ and Δ*CV*_*i*_^*Y*^ represent the variations of the corresponding coefficients of variation of the *i*th input parameter and the output parameter, respectively.

### 2.3. Uncertainty Analysis

A Monte Carlo simulation was utilized to study the impact of the simultaneous changes in the parameters on the model results, that is, the uncertainty of the model. Based on an analysis of the collected parameter values, all of the parameters except for temperature (*T*) were assumed to follow the lognormal distribution [22].

A total of 2200 Monte Carlo simulation runs were conducted. Both static and dynamic parameters with higher sensitivity coefficients were selected and the original values were retained for the remaining parameters in the simulation process. The geometric mean and standard deviation could be calculated for static parameters with multiple values. Conversely, if only one value was obtained, the corresponding coefficients of variation for the parameters were assigned using values based on the literature [2, 22]. For dynamic parameters, the monthly geometric mean and standard deviation were calculated from hourly or daily data. When only monthly data were available, the coefficients of variation were manually assigned. Each run was implemented with values for each parameter that were randomly selected in the range of the mean ± standard deviation. Semi-interquartile ranges for the monthly model output were obtained for the uncertainty analysis.

## 3. Results and Discussion

### 3.1. Concentrations of *α*-HCH in Various Media and Models Validation

**Figure 2:**
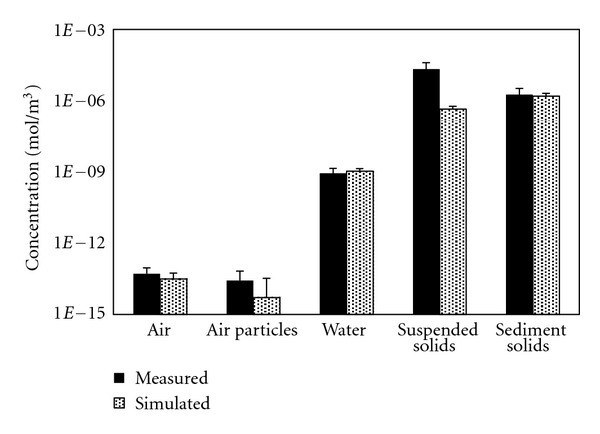
Comparison between the simulated and measured *α*-HCH concentrations in the air, water, and sediment of Lake Chaohu. The error bars included in this figure represent the standard deviations.

The simulated annually average concentrations of *α*-HCH in the air, water, and sediment are shown in Figure 2 and were found to be in agreement with the measured data. The differences in the main phases were 0.21, 0.06, and 0.07 logarithmic units for the air, water, and sediment, respectively, which were all within 0.5 log units during the simulation. The air concentrations were underestimated, which might be due to various factors. On the other hand, in addition to the uncertainty of the model, the underestimation in the sediment may be due to the fact that the samples were collected in 2008, while the model simulation period was 2010-2011. The overestimation of the concentration in the water may have been due to the absence of a biological phase. Aquatic organisms, especially plankton, can substantially affect the fate of persistent organic pollutants (POPs) in the water environment [23]. It can be observed that the *α*-HCH concentration in the sediment particles was much higher than that in the atmosphere or in the water bodies. It was concluded that sediment is an important sink of *α*-HCH [24].

The simulation results for the atmospheric particulates and the suspended solids in the water were not satisfactory. The differences between the measured and simulated data are 0.6 and 1.69 orders of magnitude, respectively. The underestimation of the *α*-HCH concentration in atmospheric particulates may be associated with the underestimation of the organic carbon content or the volume ratio of the atmospheric particulates. It was always acceptable if the deviations between the simulated and observed data were less than 0.5 or 0.7 orders of magnitude for multimedia fugacity model [2]. Thereby, the results in the air particles should be acceptable. The underestimation of the *α*-HCH concentration in the suspended solids in the water may be related to similar processes as those that caused the overestimation in the water.

**Figure 3:**
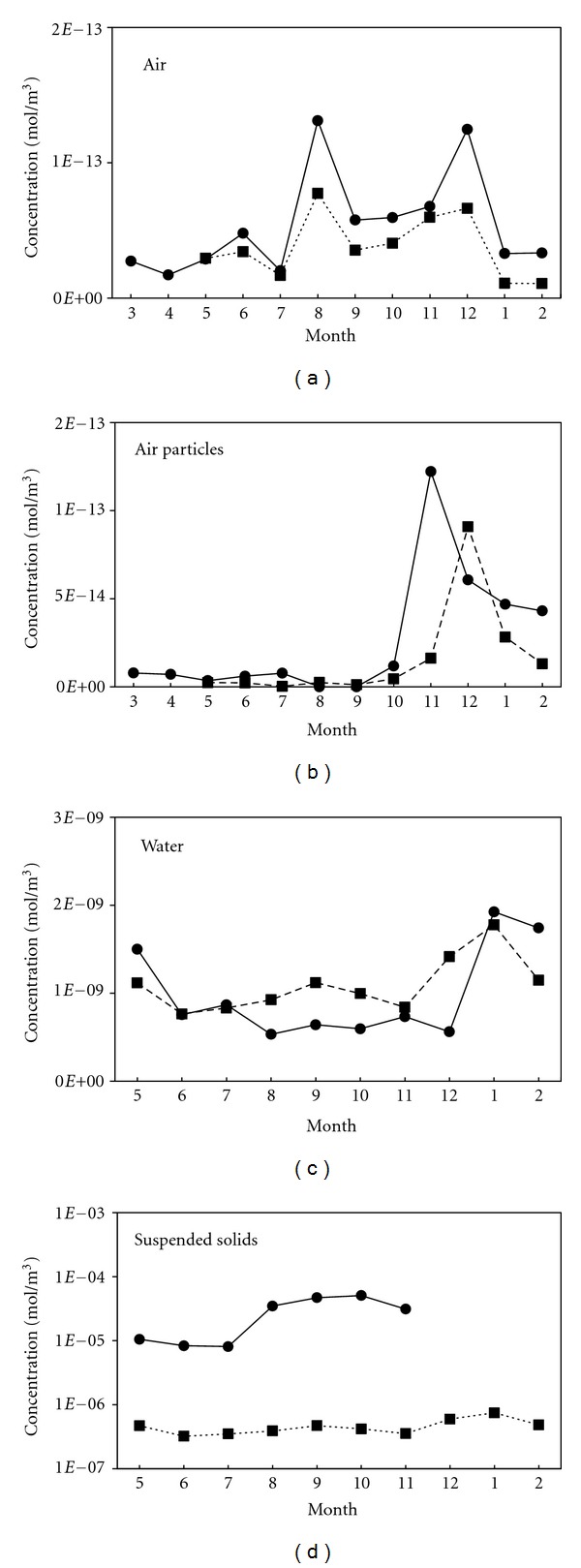
Seasonal variations of *α*-HCH concentrations in the various environmental media. Both the simulated (dashed) and measured (solid) concentrations are presented for model validation.

The simulation results regarding the monthly *α*-HCH concentration in different compartments are shown in Figure 3. The model output of the *α*-HCH concentrations in the atmosphere and the atmospheric particles was consistent with the measured values. However, the *α*-HCH concentration in the atmospheric particles peaked in November according to the measured values, while the calculated value peaked in December, which corresponded to the peak of gaseous *α*-HCH concentration but failed to capture the November peak. This discrepancy may be due to higher concentrations of *α*-HCH in the remote input of atmospheric particulate matter in November. The specific mechanisms underlying this difference require further study. 

Gaseous *α*-HCH concentrations in the summer and winter, notably in August and December, were higher than in other seasons. Ridal et al. [25] also observed relatively high concentrations of gaseous *α*-HCH in Lake Ontario in August. The most likely cause of higher *α*-HCH concentrations in the summer may be the higher temperatures in summer months [26], which favor volatilization. High values in the winter may be due to remote inputs from the atmosphere [27]. Haugen et al. [28] suggested that when the regression coefficient *R* ^2^ for ln⁡*P* and 1/*T* is high, local gaseous *α*-HCH is mainly derived from surface volatilization. Otherwise, remote input is typically the dominant source of gaseous *α*-HCH. In this study, the regression coefficient for ln⁡*P* and 1/*T* was 0.004, indicating that the gaseous *α*-HCH in Lake Chaohu was influenced to a greater extent by remote input than by lake volatilization. Agricultural land accounted for 61.12% of the total land area in the Lake Chaohu watershed [13]. Consequently, large amounts of *α*-HCH residues remain in the soils. After volatilization, the *α*-HCH is able to be transported to the lake by air advection. In addition, there was a slight decline of gaseous *α*-HCH in July (Figure 3), which corresponded to a marked increase in the wet deposition flux (*T*_12*r*_) during this period. The reason for the slight decline may be increased precipitation. It can be concluded that both temperature and precipitation are key factors affecting gaseous *α*-HCH. This conclusion was quantitatively verified using the sensitivity analysis. It is worth noting that wet deposition (*T*_12*w*_) was higher in the summer, particularly in August, and lower during the other seasons. In contrast, dry deposition (*T*_12*p*_) was higher in the winter, notably in December, and lower during the other seasons.

*α*-HCH in atmospheric particulate matter was lower in the summer and higher in the winter. The primary reason behind this difference may be that as the temperature rises in the summer, the gas-solid balance of *α*-HCH in the air shifts toward the gaseous phase. The situation is opposite in the winter [15]. In addition, the atmospheric particulate matter content in the summer is lower due to a decrease in the remote inputs when compared to winter.

The measured and simulated values of *α*-HCH in the water were also in good agreement. The model captured the high value in the winter and the variation in the other seasons, which was also consistent with the data Ridal et al. [25] observed in Lake Ontario. The peak in the winter values may be attributed to several causes. First, although the winter temperatures are lower, leading to reduced water fugacity capacity [15], the precipitation and water inflow are also lower in the winter, resulting in a significant decrease in water levels, which may cause a concentration effect. Furthermore, the gas-to-water diffusion process flux (*T*_12*d*_) is higher in the winter, which may also be important. Conversely, lower concentrations were simulated in the summer and the autumn. A noticeable decline occurred in June, which may be due to the dilution effect caused by the rising water levels and elevated water-to-air diffusion (*T*_12*d*_) caused by increasing temperatures. The *α*-HCH concentrations in water begin to be overestimated from August through December, which coincides with an observed increase in the seasonal distribution of cyanobacteria in Lake Chaohu [29]. In addition, the calculated value of the *α*-HCH concentration in the suspended solids was much less than the measured value by a factor of more than one order of magnitude every month. It can be speculated that disregarding aquatic organisms, particularly the phytoplankton phase, can lead to a significant deviation between the measured data and simulation results. Phytoplankton uptake is strongly affecting the fate of persistent organic pollutants (POPs) in aquatic environments [23], which was not included in this model. Only absorption by the organic matter in the suspended solids was considered in the model. Dachs et al. [23] proposed a model combining POPs in the air-water exchange and phytoplankton absorption processes. However, currently there is no data on the parameters of HCH exchange between water and phytoplankton [30, 31]. A modification in the model structure and further research are needed in the future.

The annual averages of the sampled values of *α*-HCH content in the sediment particles were consistent with the simulated results. Similar seasonal variations in the water bodies were obtained, showing the trends of higher values in the summer and lower values in the winter. With smaller seasonal changes, the *α*-HCH content in the sediment was relatively stable compared to that in the water.

### 3.2. Transfer Fluxes of *α*-HCH between Compartments

**Figure 4:**
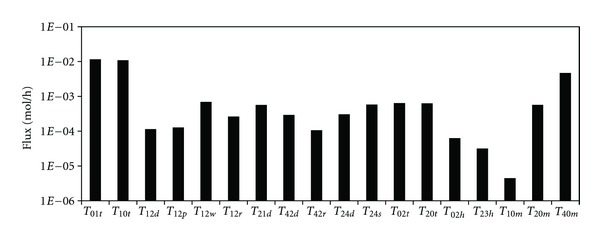
*α*-HCH fluxes in and out of the Lake Chaohu area and between the adjacent compartments. *T*_01*t*_: air advection flows into the area; *T*_10*t*_: air advection flows out of the area; *T*_12*d*_: diffusion from air to water; *T*_12*p*_: dry deposition from air to water; *T*_12*w*_: wet deposition from air to water; *T*_12*r*_: rain scavenging; *T*_21*d*_: diffusion from water to air; *T*_42*d*_: diffusion from sediment to water; *T*_42*r*_: resuspension from sediment to water; *T*_24*d*_: diffusion from water to sediment; *T*_24*s*_: sedimentation from water to sediment; *T*_02*t*_: water advection flows into the area; *T*_20*t*_: water advection flows out of the area; *T*_02*h*_: locative wastewater discharge; *T*_23*h*_: industry and agriculture water usage; *T*_10*m*_: degradation in air; *T*_20*m*_: degradation in water; *T*_40*m*_: degradation in sediment.

As shown in Figure 4, the net input of *α*-HCH into the Lake Chaohu environment is approximately 0.115 mol/h (approximately 0.294 t/a), while the net output is 0.162 mol/h (approximately 0.412 t/a). It can be observed that the *α*-HCH content in the Lake Chaohu watershed is diminishing. The atmospheric advection input was found to be the main source (*T*_01*t*_) (0.278 t/a), which corresponded to the atmospheric advection output (*T*_10*t*_) (0.277 t/a). By contrast, the *α*-HCH input from water inflows was very small (0.016 t/a). An important output was the degradation in the sediments (0.119 t/a), which accounted for 89.05% of the total degradation in the environment, while the degradation in the water was 0.015 t/a, which accounted for 10.86% of the total degradation.

**Figure 5:**
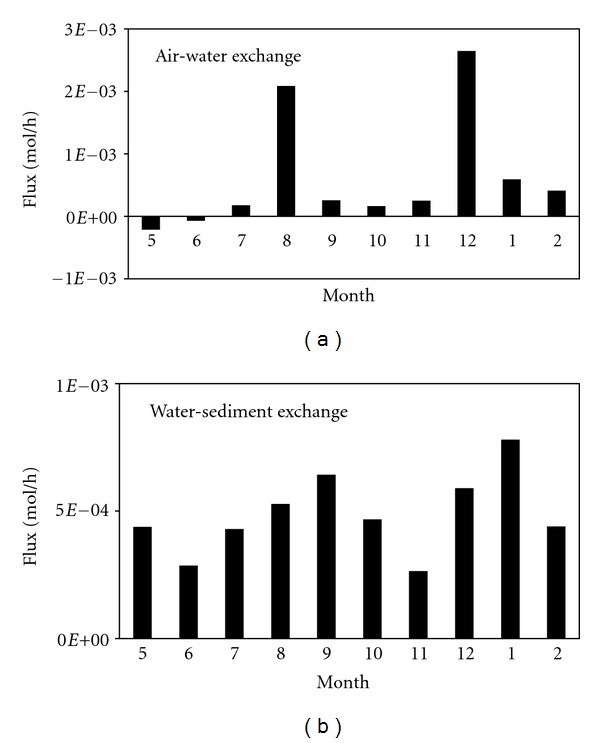
*α*-HCH fluxes over the air-water interface (a) and the water-sediment interface (b). The positive values indicate net inputs from air to water or from water to sediment.

For interface processes, the atmospheric input to the water was 0.030 t/a, and the dominant process of atmospheric input to the water was precipitation scavenging (*T*_12*w*_), which accounted for 57.80% of the gas-to-water flux. The flux of diffusion from the water to the atmosphere (*T*_21*d*_) was 0.014 t/a. Therefore, there was an annual net input from the atmosphere to the water. The seasonal variations in the air-water exchange were shown in Figure 5. There was a net volatilization from the water into the atmosphere in May, which was consistent with the results obtained by Taihu [32]. During the other seasons, however, there is a net input from the atmosphere to the water, which is the converse of the results observed in Lake Taihu. The main cause of this difference may be that the research in Lake Taihu did not include deposition from air to water. It is also worth noting that the *α*-HCH concentrations in the Lake Taihu atmosphere and water are 32 ± 28 pg/m^3^ and 1887 ± 1372 pg/L, respectively, while in Lake Chaohu, the corresponding concentrations are 16 ± 11 pg/m^3^ and 423 ± 395 pg/L, which are 50.0% and 22.4% of the values of Lake Taihu, respectively. The lower *α*-HCH concentration in the water of Lake Chaohu may be due to historically lower HCH pesticide usage. The results are also opposite from the findings for Lake Ontario [25]. Ridal et al. [25] proposed that, due to a reduction in the atmospheric concentration, the *α*-HCH flux in Lake Ontario has shifted from net settlement to net volatilization when compared with the years prior to 1990. For Chaohu, however, due to a reduction in the water *α*-HCH concentration, the air-water interface may have still been net settlement. Therefore, despite the net volatile flux in the summer, the annual net flux is from the gas to the water.

The flux from the water to the sediment was 0.022 t/a, and sedimentation (*T*_24*s*_) accounted for 65.49% of this flux. In addition, the flux from the sediment to the water was 0.010 t/a, and diffusion flux (*T*_42*d*_) accounted for 73.28% of this flux. There was a net input from the water to the sediment (Figure 5). Although the sediment resuspension flux was 0.003 t/a, which accounted for 26.72% of the flux from the sediment to the water, this flux still reflects the strong resuspension process in Lake Chaohu [13].

In the sensitivity analysis, those parameters related to relatively important processes will always be observed with higher sensitivity (see Section 3.3).

### 3.3. Sensitivity Analysis

**Figure 6:**
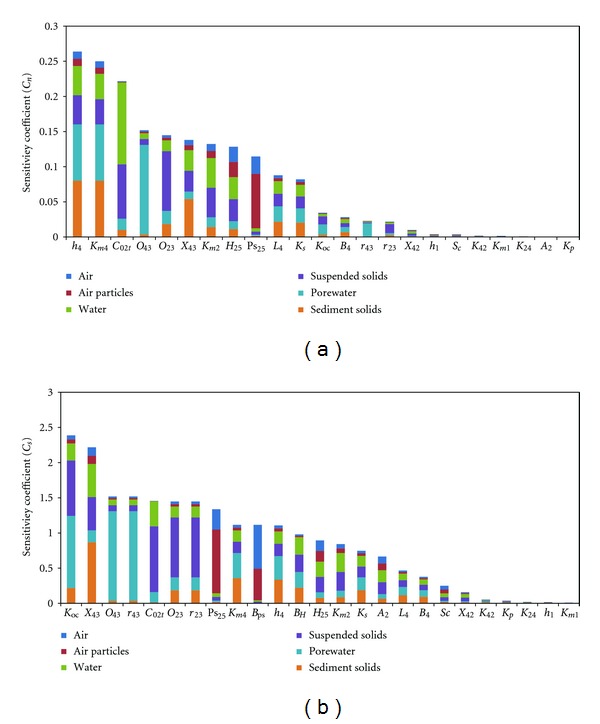
Coefficients of sensitivity of the calculated concentrations of the environmental compartments to the input static parameters with (*C*_*n*_) (a) and without (*C*_*s*_) (b) CV normalization.

For the static parameters, the sensitivity coefficients changed significantly after correction with the coefficients of variation (Figure 6). Thus, despite the high sensitivities regarding *K*_oc_, *r*_23_, *r*_43_, *B*_ps_, *B*_*H*_,*A*_2_, and Sc, the corrected sensitivity coefficients for those parameters with lower variability were significantly reduced, such that these eight parameters were considered to be insensitive. The sensitivity reductions in *K*_oc_ and *A*_2_ were also observed by Cao et al. [2]. In contrast, due to higher variability, the sensitivity coefficients of *h*_4_, *k*_*m*4_, *k*_*m*2_, and *L*_4_ increased after correction, and they were found to be important parameters. *h*_4_ is related to sediment volume, and sediment is found as the sink for *α*-HCH in lakes; *k*_*m*4_ is directly related to the degradation of *α*-HCH in the sediments, which has been found to be the most important degradation process in the environment (Section 3.2). Thus, the two static parameters exerted considerable influence on the model results. *L*_4_ and *k*_*m*2_ become more important parameters due to their high variability. Other parameters, including *C*_02*t*_, *O*_23_, *O*_43_, *X*_43_, Ps_25_, and *H*_25_, had relatively similar high sensitivity coefficients before and after correction. *C*_02*t*_ strongly affects the *α*-HCH content in the water and suspended matter. *O*_23_ and *O*_43_ determine the adsorption capacity of the particles in the suspended solids and sediments, while *X*_43_ is related to the amount of sediment adsorption. Therefore, these parameters exert a great influence on the model output. Ps_25_ determines the fugacity capacity of the atmospheric particulates [33], and *H*_25_ plays a decisive role in the fate of POPs in the environment [34]. Although the variability of these two parameters is negligible, the collected values in this study are based on the results from different time periods using different methods. Therefore, the sensitivities of these two parameters remain high after the correction.

Each of the parameters has a different influence on the various environmental compartments. For example, *k*_*m*4_ has a higher sensitivity coefficient for the sediment than for the water or atmosphere, while *k*_*m*2_ has the highest sensitivity coefficient for the water. Overall, the average values of *C*_*n*_ for the air, water, and sediment were 1.17%, 2.78%, and 3.42%, respectively. Although water contains the most parameters among the three main phases [10], the sediment serves as an important sink for *α*-HCH and is influenced by all of the parameters to a greater extent than either the air or the water.

**Figure 7:**
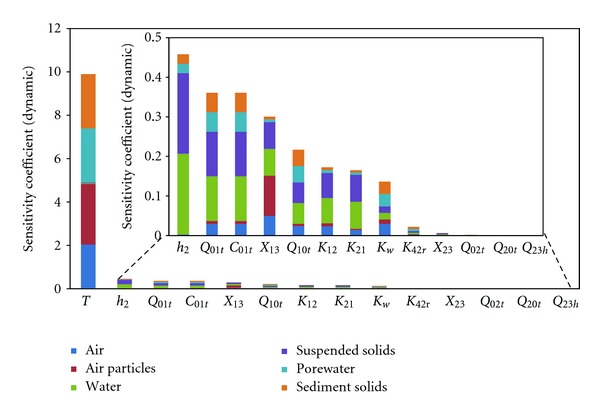
Dynamic coefficients of sensitivity of the calculated concentrations of the environmental compartments to the input dynamic parameters.

The dynamic sensitivity coefficients (SCV) are shown in Figure 7. The model output was much more sensitive to temperature (*T*) than to the other parameters because temperature had very strong effects on Ps and *H*, the two important parameters in the model. Consequently, temperature played a decisive role in the distribution of *α*-HCH between the gaseous and particulate phases as well as between the air and water [24]. In addition, *h*_2_, *Q*_01*t*_, *Q*_10*t*_, *C*_01*t*_, *X*_13_, *K*_12_, *K*_21_, and K_*w*_ also had strong influences on the dynamic changes of the model output. *h*_2_ strongly affected the variation of *α*-HCH concentrations in the water and suspended solids; *Q*_01*t*_, *Q*_10*t*_, and *C*_01*t*_ were associated with the atmospheric advection, which was the main source of the *α*-HCH in Lake Chaohu. Thus, the seasonal variations in these three parameters also had significant impacts. Cao et al. [2] found that the parameters related to source and degradation in the fugacity model were relatively more important, which was consistent with the relatively high sensitivities of *Q*_01*t*_, *Q*_10*t*_, *C*_01*t*_, *k*_*m*4_, and *k*_*m*2_. *X*_13_ had a relatively strong influence on the seasonal changes in the concentration in the atmosphere and the water bodies as well as the particulate and suspended matter content, which is in agreement with the conclusion of the Pearl River Delta study [22]; *K*_12_, *K*_21_, and *K*_*w*_ were the main parameters influencing the air-water interface flux due to their direct impacts and significant seasonal variations, and these three parameters are also important parameters generally. In addition, due to the insignificant effect of water inflows on the model, parameters such as *Q*_02*t*_, *Q*_20*t*_, and *Q*_23*h*_ had little effect on the variability of the model output. Without considering the biological phase, the importance of *X*_23_ was also reduced. The low sensitivity coefficient of *K*_42*r*_ was due to the corresponding low resuspension flux.

### 3.4. Uncertainty Analysis

**Figure 8:**
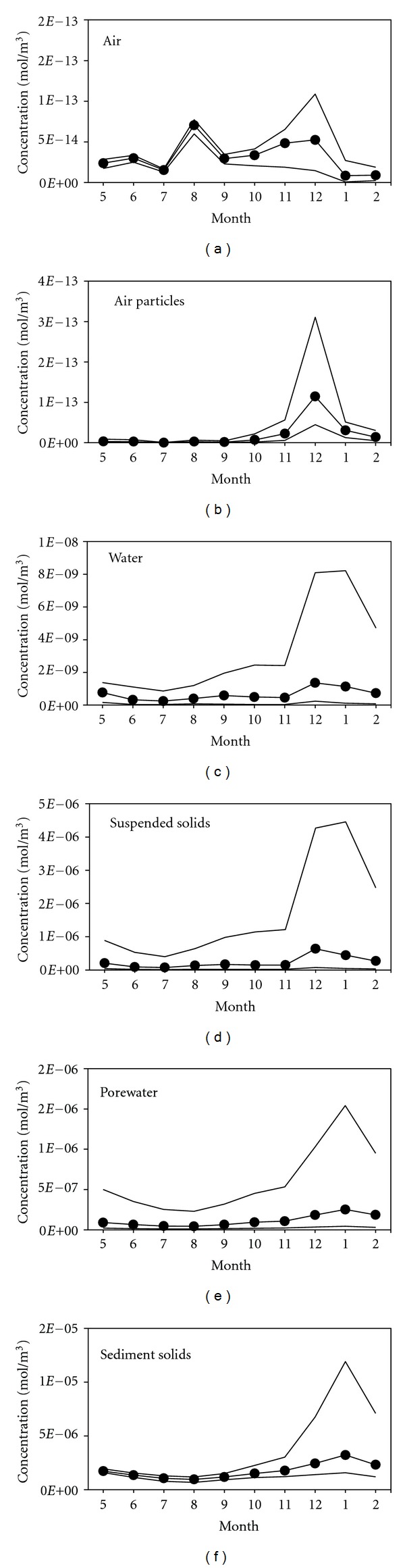
Uncertainties of the predicted seasonal variations of *α*-HCH concentrations in various environmental media. The results are presented as median values (lines with dots) and semi-interquartile ranges (solid lines).

The results of the uncertainty analysis for each phase are shown in Figure 8. It was found that the uncertainty of the model was relatively small from May to September, as represented by the small semi-interquartile ranges of the Monte Carlo simulation results. The uncertainty of the model output began to increase in October and peaked in December or January. This increase was attributed to our finding that from October to December, the coefficients of variation in the gas-water diffusion rate (*K*_12_ and *K*_21_) significantly increased, leading to an increase of variation in the air-water diffusion flux. This also contributed to a significant increase in the uncertainty of the other phases. Lang et al. [22] similarly found that the coefficient of variability of diffusion is associated with wide variability in the gaseous PAHs concentrations. The rates of diffusion across the gas-water interface (*K*_12_ and *K*_21_) were related to wind speed and water depth, and the coefficient of variation of water depth (*h*_2_) did not increase during October–December. It can be speculated that elevated variation in the wind speed in this period causes the increasing uncertainty. 

## 4. Conclusions

A dynamic quantitative water, air, and sediment interaction (QWASI) fugacity model was utilized to simulate the fate and seasonal variations of *α*-HCH in the air, water, and sediment, as well as various environmental fluxes in Lake Chaohu. The calculated and measured values of the model were in good agreement. However, disregarding the effects of aquatic organisms resulted in large deviations between the simulated and measured values of *α*-HCH in suspended solids in water. The major source of *α*-HCH in Lake Chaohu was an input from atmospheric advection, while the major environmental outputs were atmospheric advection and sediment degradation. The net annual input of *α*-HCH into the lake area was approximately 0.294 t, while the net output was approximately 0.412 t. The factors leading to the seasonal variations of *α*-HCH in various compartments were revealed. For the fluxes at the air-water interface, atmospheric inputs into the water were dominant for most of the year with the deposition processes included, while the water and sediment interface was mainly influenced by the net input from the water to the sediment. Thus, sediment is an important sink for *α*-HCH. Sensitivity analysis found that parameters of source and degradation were more important than the other parameters. The sediment was influenced more by the combined effects of the various parameters than air and water were. In addition, temperature variation had a much greater impact on the dynamics of the model output than other dynamic parameters. Uncertainty analysis showed that the model uncertainty was relatively low, especially in the first half of the simulation period. Due to the increase in the gas-water diffusion flux variability, uncertainty of the model significantly increased for all of the compartments.

## Supplementary Material

In Supplementary Material, we provide three tables illustrating environmental parameters (Table S1), mass transfer kinetic and physical-chemical parameters (Table S2) and definitions of the transfer and transformation processes (Table S3) for the model in this study.

## Figures and Tables

**Table 1:** Volume and properties of the phases and sub-phases.

Main phase	Area (m^2^)	Depth (m)	Organic carbon (%)	Subphases and volume fraction (*X*_*ij*_)		
				Air (1)	Water (2)	Solid particles (3)
Air (1)	7.5810*E* + 08	1.0000*E* + 02	—	1.0000*E* + 00	—	7.6278*E* − 11^a^
Water (2)	7.5810*E* + 08	3.0124*E* + 00^a^	1.6700*E* − 01	—	1.0000*E* + 00	1.2631*E* − 05^a^
Sediment (3)	7.5810*E* + 08	1.0000*E* − 01	4.6077*E* − 03	—	7.0000*E* − 01	3.0000*E* − 01

a: annually average value; seasonal data were available in simulation.

## References

[1] Stockholm Convention on persistent organic pollutants (SCPOPs) http://chm.pops.int/Programmes/NewPOPs/Publications/tabid/695/ctl/Download/mid/2784/language/en-US/Default.aspx?id=2.

[2] Cao H, Tao S, Xu F (2004). Multimedia fate model for hexachlorocyclohexane in Tianjin, China. *Environmental Science and Technology*.

[3] Mackay D (2001). *Multimedia Environmental Models: The Fugacity Approach*.

[4] Mackay D (1979). Finding fugacity feasible. *Environmental Science and Technology*.

[5] Mackay D, Paterson S (1981). Calculating fugacity. *Environmental Science and Technology*.

[6] Mackay D, Paterson S (1982). Fugacity revisited. *Environmental Science and Technology*.

[7] Wania F, Mackay D (1995). A global distribution model for persistent organic chemicals. *Science of the Total Environment*.

[8] Liu Z, Quan X, Yang F (2007). Long-term fate of three hexachlorocyclohexanes in the lower reach of Liao River basin: dynamic mass budgets and pathways. *Chemosphere*.

[9] Tao S, Cao H, Liu W (2003). Fate modeling of phenanthrene with regional variation in Tianjin, China. *Environmental Science and Technology*.

[10] Tao S, Yang Y, Cao HY (2006). Modeling the dynamic changes in concentrations of *γ*- hexachlorocyclohexane (*γ*-HCH) in Tianjin region from 1953 to 2020. *Environmental Pollution*.

[11] Mackay D, Joy M, Paterson S (1983). A quantitative water, air, sediment interaction (QWASI) fugacity model for describing the fate of chemicals in lakes. *Chemosphere*.

[12] Paasivirta J, Sinkkonen S, Mikkelson P, Rantio T, Wania F (1999). Estimation of vapor pressures, solubilities and Henry’s law constants of selected persistent organic pollutants as functions of temperature. *Chemosphere*.

[13] Tu QY, Gu DX, Yi CQ, Xu ZR, Han GZ (1990). *The Researches on the Lake Chaohu Eutrophication*.

[14] China Meteorological Data Sharing Service System (CMDSSS) http://www.cma.gov.cn/2011qxfw/2011qsjgx/index.htm.

[15] Carlson DL, Basu I, Hites RA (2004). Annual variations of pesticide concentrations in great lakes precipitation. *Environmental Science and Technology*.

[16] Southworth GR (1979). The role of volatilization in removing polycyclic aromatic hydrocarbons from aquatic environments. *Bulletin of Environmental Contamination and Toxicology*.

[17] Mackay D, Paterson S (1991). Evaluating the multimedia fate of organic chemicals: a level III fugacity model. *Environmental Science and Technology*.

[18] Liu WX, He W, Qin N (2012). Residues, distributions, sources and ecological risks of OCPs in the water from Lake Chao, China. *The Scientific World Journal*.

[19] Ouyang HL, He W, Qin N (2012). Levels temporal-spatial variations and sources of organochlorine pesticides in ambient air of Lake Chaohu, China. *The Scientific World Journal*.

[20] Wang Y, He W, Qin N, He QS (2012). Residual levels and ecological risks of organochlorine pesticides in surface sediments from Lake Chaohu. *Acta Scientiae Circumstantiae*.

[21] Morris MD (1991). Factorial sampling plans for preliminary computational experiments. *Technometrics*.

[22] Lang C, Tao S, Wang X, Zhang G, Li J, Fu J (2007). Seasonal variation of polycyclic aromatic hydrocarbons (PAHs) in Pearl River Delta region, China. *Atmospheric Environment*.

[23] Dachs J, Eisenreich SJ, Baker JE, Ko FC, Jeremiason JD (1999). Coupling of phytoplankton uptake and air-water exchange of persistent organic pollutants. *Environmental Science and Technology*.

[24] Walker K, Vallero DA, Lewis RG (1999). Factors influencing the distribution of lindane and other hexachlorocyclohexanes in the environment. *Environmental Science and Technology*.

[25] Ridal JJ, Kerman B, Durham L, Fox ME (1996). Seasonally of air-water fluxes of hexachlorocyclohexanes in lake Ontario. *Environmental Science and Technology*.

[26] Zheng X, Chen D, Liu X (2010). Spatial and seasonal variations of organochlorine compounds in air on an urban-rural transect across Tianjin, China. *Chemosphere*.

[27] Li YF, Macdonald RW, Jantunen LMM, Harner T, Bidleman TF, Strachan WMJ (2002). The transport of *β*-hexachlorocyclohexane to the western Arctic Ocean: a contrast to *α*-HCH. *Science of the Total Environment*.

[28] Haugen JE, Wania F, Ritter N, Schlabach M (1998). Hexachlorocyclohexanes in air in southern Norway. Temporal variation, source allocation, and temperature dependence. *Environmental Science and Technology*.

[29] Xie P (2009). *Reading about the Histories of Cyanobacteria, Eutrophication and Geological Evolution in Lake Chaohu*.

[30] Berrojalbiz N, Dachs J, Del Vento S (2011). Persistent organic pollutants in mediterranean seawater and processes affecting their accumulation in plankton. *Environmental Science and Technology*.

[31] del Vento S, Dachs J (2002). Prediction of uptake dynamics of persistent organic pollutants by bacteria and phytoplankton. *Environmental Toxicology and Chemistry*.

[32] Qiu X, Zhu T, Wang F, Hu J (2008). Air-water gas exchange of organochlorine pesticides in Taihu Lake, China. *Environmental Science and Technology*.

[33] Mackay D, Paterson S, Schroeder WH (1986). Model describing the rates of transfer processes of organic chemicals between atmosphere and water. *Environmental Science and Technology*.

[34] Odabasi M, Cetin B, Demircioglu E, Sofuoglu A (2008). Air-water exchange of polychlorinated biphenyls (PCBs) and organochlorine pesticides (OCPs) at a coastal site in Izmir Bay, Turkey. *Marine Chemistry*.

