# Supplementary material for: Simulation of the Fate and Seasonal Variations of α-Hexachlorocyclohexane in Lake Chaohu Using a Dynamic Fugacity Model
Source: ScientificWorldJournal. 2012 Dec 18;2012:691539. doi: 10.1100/2012/691539 (PMC3539339; doi:10.1100/2012/691539)
Supplement: Supplementary file 1 — In Supplementary Material, we provide three tables illustrating environmental parameters (Table S1), mass transfer kinetic and physical-chemical parameters (Table S2) and definitions of the transfer and transformation processes (Table S3) for the model in this study. [file 691539.f1.docx]

**Supplementary Materials for**

**Simulation of the fate and seasonal variation of α-HCH in Lake Chaohu using a dynamic fugacity model**

Xiangzhen Kong, Wei He, Ning Qin, Qishuang He, Bin Yang, Huiling Ouyang, Qingmei Wang, Chen Yang, Yujiao Jiang, Fuliu Xu*

MOE Laboratory for Earth Surface Processes, College of Urban & Environmental Sciences, Peking University, Beijing 100871, China

*Corresponding author. Tel: (86) 10-62751177; Fax: (86) 10-62751187; Email: [xufl@urban.pku.edu.cn](mailto:xufl@urban.pku.edu.cn).

**The supplementary materials include three tables. The titles for these tables are as follows:**

Table S1 Environmental parameters for the model

Table S2 Mass transfer kinetic and physical-chemical parameters for the model

Table S3 Definitions of the transfer and transformation processes

Table S1 Environmental parameters for the model

| Symbol | Unit | Parameters | Reference | Data Numbers | Allocate mean | Allocate SD | Geometric mean | Geometric SD |
| --- | --- | --- | --- | --- | --- | --- | --- | --- |
| A_2_ | m^2^ | Interface areas of air/water | 1,2 | 2 | 7.58E+08 | 2.69E+06 | 7.58E+08 | 1.00E+00 |
| h_1_ | m | Thickness of air | 1 | 1 | 1.00E+02 | -^b^ | 1.00E+02 | 1.00E+00 |
| h_2_^a^ | m | Depth of water | 3 | - | - | - | - | - |
| h_4_ | m | Thickness of sediment | 1 | 1 | 1.00E-01 | - ^b^ | 1.00E-01 | 5.78E-01 |
| X_13_^a^, X_23_ | v/v | Volume fractions of solids in air and water | 4, Note A | - | - | - | - | - |
| X_43_ | v/v | Volume fractions of solids in sediment | 4 | 1 | 3.00E-01 | - ^b^ | 3.00E-01 | 1.08E+00 |
| X_42_ | v/v | Volume fractions of water in sediment | 4 | 1 | 7.00E-01 | - ^b^ | 7.00E-01 | 1.02E+00 |
| O_23_ | % | Contents of organic carbon in solids in water | 4 | 1 | 1.67E-01 | - ^b^ | 1.67E-01 | 1.20E+00 |
| O_43_ | % | Contents of organic carbon in solids in sediment | 1,2,5, Note A | 61 | 5.27E-03 | 2.92E-03 | 4.61E-03 | 1.72E+00 |
| ρ_23_ | t/m^3^ | Densities of solids in water | 1 | 1 | 1.50E+00 | - ^b^ | 1.50E+00 | 1.06E+00 |
| ρ_43_ | t/m^3^ | Densities of solids in sediment | 1 | 1 | 2.76E+00 | - ^b^ | 2.76E+00 | 1.16E+00 |
| Q_01t_^a^, Q_10t_^a^ | m^3^/h | Air advection flow in and out of the area | Calculated | - | - | - | - | - |
| Q_02t_^a^, Q_20t_^a^ | m^3^/h | Water advection flow in and out of the area | Calculated | - | - | - | - | - |
| Q_02h_^a^ | m^3^/h | Rate of local wastewater discharge | 1 | - | - | - | - | - |
| C_1_^a^ | mol/m^3^ | α-HCH concentration in air | Note A | - | - | - | - | - |
| C_02t_ | mol/m^3^ | α-HCH concentration in water advection flow | 1,6 | 1 | 1.34E-08 | - ^b^ | 1.34E-08 | 1.63E+00 |
| C_02h_ | mol/m^3^ | α-HCH concentration in wastewater | 1 | 1 | 1.56E-08 | - ^b^ | 1.56E-08 | 1.82E+00 |
| T^a^ | K | Local average temperature | 7 | - | - | - | - | - |

References: [1]Tu, 1990; [2]Yin, 2011; [3] AHTIS（<http://61.191.22.154/yc_web/yc_index_frame.aspx>）. [4] Mackay, 2001; [5] Zhou et al., 2007; [6] Zhang, 2009. [7] CMDSSS（<http://www.cma.gov.cn/2011qxfw/2011qsjgx/index.htm>）.

Note A: Measured in Labotary.

^a^ dynamic parameters (Values not shown)

^b^ One value only; Geometric SD assighed manually (Log-normal distribution assumed)

[1] Q.Y. Tu, D.X. Gu, C.Q. Yi, Z.R. Xu and G.Z. Han, 1990. *The Researches on the Lake Chaohu Eutrophication.* , Publisher of University of Science and Technology of China, Hefei (in Chinese).

[2] F.C. Yin, 2011. *A study on evaluation and control instruments of Chao lake eutrophication*, China Environmental Science Press Beijing (in Chinese).

[3] Anhui Hydrological Telemetry Information System (AHTIS), 2010-2011. Hourly water level report. <http://61.191.22.154/yc_web/yc_index_frame.aspx>

[4] D. Mackay, 2001. Multimedia Environmental Models: The Fugacity Approach,second ed. , Lewis Publishers, NewYork, USA.

[5] Z.H. Zhou, C.Q. Liu, J. Li and Z.Z. Zhu, "Record of Ecosystem Evolvement Processes Provided by δ13Corg and δ15 N Values inChaohu Lake Sediments," *ENVIRONMENTAL SCIENCE,* vol. 28, no. 6, pp. 1338-1343 (in Chinese), 2007.

[6] M Zhang, 2009. Distribution characteristic and assessment of typical persistent organic pollutions-Organochlorine pesticides in water of Chaohu Lake watershed. Anhui Agriculture University, Hefei (in Chinese).

[7] China Meteorological Data Sharing Service System (CMDSSS), 2010-2011. Daily Meteorological Data. <http://www.cma.gov.cn/2011qxfw/2011qsjgx/index.htm>

Table S2 Mass transfer kinetic and physical-chemical parameters for the model

| Symbol | Unit | Parameters | Reference | Data Numbers | Allocate mean | Allocate SD | Geometric mean | Geometric SD |
| --- | --- | --- | --- | --- | --- | --- | --- | --- |
| P_S_ | Pa | Local vapor pressure | 1-6 | 6 | 4.92E-02 | 1.08E-01 | 9.01E-03 | 1.50E+00 |
| R | Pa⋅m^3^/mol⋅K | The gas constant | 1 | 1 | 8.31E+00 | 0.00E+00 | 8.31E+00 | 1.00E+00 |
| F_25_ | - | Fugacity ratio at 25℃ | 7 | 1 | 1.21E-02 | -^b^ | 1.21E-02 | 1.00E+00 |
| H | Pa⋅m^3^/mol | Henry’s constant | 1,3,5,6 | 4 | 6.12E-01 | 4.18E-02 | 6.11E-01 | 1.07E+00 |
| B_F_ | - | Fugacity ratio temperature correction factor | 7 | 1 | 1.62E+03 | -^b^ | 1.62E+03 | 1.00E+00 |
| B_H_ | - | Henry’s law constant temperature correction factor | 7 | 1 | 1.71E+03 | -^b^ | 1.71E+03 | 1.00E+00 |
| BP_S_ | - | Saturation vapor pressure temperature correction factor | 7 | 1 | 4.95E+03 | -^b^ | 4.95E+03 | 1.00E+00 |
| K_OC_ | m^3^/t,1/h | Adsorption coefficient | 1,3,5 | 3 | 1.69E+03 | 1.86E+02 | 1.68E+03 | 1.11E+00 |
| K_m1_ | - | Degradation rate of α-HCH in air | 1,2,5,8,9 | 4 | 2.43E-03 | 3.70E-03 | 1.09E-03 | 3.86E+00 |
| K_m2_ | - | Degradation rate of α-HCH in water | 1,2,5,10 | 4 | 3.17E-04 | 3.33E-04 | 2.18E-04 | 2.63E+00 |
| K_m4_ | - | Degradation rate of α-HCH in sediment | 1,2,3,5,10 | 5 | 4.87E-04 | 7.37E-04 | 1.22E-04 | 5.52E-01 |
| B_1_ | m^2^/h | Molecular diffusivities in air | 2,10,11,12 | 4 | 2.49E-02 | 1.03E-02 | 2.36E-02 | 1.44E+00 |
| B_2_ | m^2^/h | Molecular diffusivities in water | 2,10,11,12 | 4 | 2.86E-06 | 1.17E-06 | 2.68E-06 | 1.54E+00 |
| B_4_ | m^2^/h | Molecular diffusivities in sediment | 2,12 | 2 | 1.43E-05 | 1.09E-05 | 1.20E-05 | 2.34E+00 |
| K_12_^a^ | m/h | Air-side molecular transfer coefficient over water | Calculated | - | - | - | - | - |
| K_21_^a^ | m/h | Water-side molecular transfer coefficient over air | Calculated | - | - | - | - | - |
| K_24_ | m/h | Water-side molecular transfer coefficient over sediment | 2 | 1 | 1.00E-02 | -^b^ | 1.00E-02 | 1.17E+00 |
| K_42_ | m/h | Water-side molecular transfer coefficient over sediment | 10,14 | 1 | 5.39415E-06 | -^b^ | 5.39E-06 | 1.52E+00 |
| K_42r_^a^ | m/h | Sediment resuspension rate | Calculated | - | - | - | - | - |
| L_4_ | m | Diffusion path lengths in sediment | 2 | 2 | 3.50E-02 | 2.12E-02 | 3.16E-02 | 1.91E+00 |
| K_P_ | m/h | Dry deposition velocity | 2,10 | 2 | 1.04E+01 | 5.66E-01 | 1.04E+01 | 1.06E+00 |
| K_S_ | m/h | Water sedimentation rates | 14,15 | 5 | 3.62E-06 | 4.34E-06 | 1.66E-06 | 4.32E+00 |
| K_w_^a^ | m/h | Wet deposition velocity | 16 | - | - | - | - | - |
| S_c_ | - | Scavenging Ratio | 2,10 | 2 | 1.34E+05 | 9.33E+04 | 1.17E+05 | 2.14E+00 |

References: [1] Cao,et al., 2007; [2]Mackay D., 2001; [3] Cao et al., 2003; [4]Cao et al., 2005; [5]Ao et al., 2009; [6]Dong et al., 2009; [7] Paasivirta et al.,1999; [8] Prinn et al., 2001; [9] Brubaker et al., 1998; [10] Breivik and Wania., 2002; [11] Mackay and Patterson, 1991; [12] Cao et al., 2004; [13]Tu, 1990; [14] Gu, 2007;[15] CDMSSS（<http://www.cma.gov.cn/2011qxfw/2011qsjgx/index.htm>）.

^a^ dynamic parameters (Values not shown)

^b^ One value only; Geometric SD assighed manually (Log-normal distribution assumed)

[1] H. Y. Cao, T. Liang, S. Tao and C. S. Zhang, "Simulating the temporal changes of OCP pollution in Hangzhou, China," *Chemosphere,* vol. 67, no. 7, pp. 1335-1345, 2007.

[2] D. Mackay, 2001. Multimedia Environmental Models: The Fugacity Approach,second ed. , Lewis Publishers, NewYork, USA.

[3] H.Y. CAO, J. CAO, F.L. XU and B.G. LI, "FATE AND TRANSFER FLUX OF HCHs IN TIANJIN," *ENVIRONMENTAL CHEMISTRY,* vol. 22, no. 6, pp. 548-554 (in Chinese), 2003.

[4] H.Y. Cao, T. Liang and S. Tao, "Dynamic simulation and prediction of BHC transfer and residues in Beijing during 50 years," *SCIENCE IN CHINA Ser. D Earth Sciences,* vol. 35, no. 10, pp. 980-988 (in Chinese), 2005.

[5] J. T. Ao, J. W. Chen, F. L. Tian and X. Y. Cai, "Application of a level IV fugacity model to simulate the long-term fate of hexachlorocyclohexane isomers in the lower reach of Yellow River basin, China," *Chemosphere,* vol. 74, no. 3, pp. 370-376, 2009.

[6] J. Y. Dong, H. Gao, S. G. Wang, H. J. Yao and M. Q. Ma, "Simulation of the transfer and fate of HCHs since the 1950s in Lanzhou, China," *Ecotoxicology and Environmental Safety,* vol. 72, no. 7, pp. 1950-1956, 2009.

[7] J. Paasivirta, S. Sinkkonen, P. Mikkelson, T. Rantio and F. Wania, "Estimation of vapor pressures, solubilities and Henry's law constants of selected persistent organic pollutants as functions of temperature," Chemosphere, vol. 39, no. 5, pp. 811-832, 1999.

[8] R. G. Prinn *et al.*, "Evidence for substantial variations of atmospheric hydroxyl radicals in the past two decades," *Science,* vol. 292, no. 5523, pp. 1882-1888, 2001.

[9] W. W. Brubaker and R. A. Hites, "OH reaction kinetics of gas-phase alpha- and gamma-hexachlorocyclohexane and hexachlorobenzene," *Environmental Science & Technology,* vol. 32, no. 6, pp. 766-769, 1998.

[10] K. Breivik and F. Wania, "Evaluating a model of the historical behavior of two hexachlorocyclohexanes in the Baltic sea environment," *Environmental Science* & Technology, vol. 36, no. 5, pp. 1014-1023, 2002.

[11] D. Mackay and S. Paterson, "EVALUATING THE MULTIMEDIA FATE OF ORGANIC-CHEMICALS - A LEVEL-III FUGACITY MODEL," Environmental Science & Technology, vol. 25, no. 3, pp. 427-436, 1991.

[12] H. Y. Cao et al., "Multimedia fate model for hexachlorocyclohexane in Tianjin, China," Environmental Science & Technology, vol. 38, no. 7, pp. 2126-2132, 2004.

[13] Q.Y. Tu, D.X. Gu, C.Q. Yi, Z.R. Xu and G.Z. Han, 1990. *The Researches on the Lake Chaohu Eutrophication.* , Publisher of University of Science and Technology of China, Hefei (in Chinese).

[14] C.J. Gu, 2005. Historical Sedimentary Records and Environmental Changes in Chaohu lake. East China Normal University, Shanghai.

[15] China Meteorological Data Sharing Service System (CMDSSS), 2010-2011. Daily Meteorological Data. <http://www.cma.gov.cn/2011qxfw/2011qsjgx/index.htm>

Table S3 Definitions of the transfer and transformation processes

| Symbol | Formula | Explanation |
| --- | --- | --- |
| System input: |  |  |
| T_01t_ | Q_01t_×C_01t_ | Air advection flows into the area |
| T_02t_ | Q_02t_×C_02t_ | Water advection flows into the area |
| T_02h_ | Q_02h_×C_02h_ | Locative wastewater discharge |
| System output: |  |  |
| T_10t_ | D_10t_×f_1_ | Air advection flows out of the area |
| T_20t_ | D_20t_×f_2_ | Water advection flows out of the area |
| T_10m_ | D_10m_×f_1_ | Degradation in air |
| T_20m_ | D_20m_×f_2_ | Degradation in water |
| T_40m_ | D_40m_×f_4_ | Degradation in sediment |
| T_23h_ | D_23h_×f_2_ | Industry and argriculture water usage |
| Air-water transfer: | |  |
| T_12d_ | D_12d_×f_1_ | Diffusion from air to water |
| T_21d_ | D_21d_×f_2_ | Diffusion from water to air |
| T_12p_ | D_12p_×f_1_ | Dry deposition from air to water |
| T_12w_ | D_12w_×f_1_ | Wet deposition from air to water |
| T_12r_ | D_12r_×f_1_ | Rain scavenging |
| Water-sediment transfer | |  |
| T_24d_ | D_24d_×f_2_ | Diffusion from water to sediment |
| T_42d_ | D_42d_×f_4_ | Diffusion from sediment to water |
| T_24s_ | D_24s_×f_2_ | Sedimentation from water to sediment |
| T_42r_ | D_42r_×f_4_ | Resuspension from sediment to water |
